# Improving Photoelectrochemical Properties of Anodic WO_3_ Layers by Optimizing Electrosynthesis Conditions

**DOI:** 10.3390/molecules25122916

**Published:** 2020-06-25

**Authors:** Marta Zych, Karolina Syrek, Leszek Zaraska, Grzegorz D. Sulka

**Affiliations:** Department of Physical Chemistry and Electrochemistry, Faculty of Chemistry, Jagiellonian University, Gronostajowa 2, 30-387 Krakow, Poland; zych@chemia.uj.edu.pl (M.Z.); zaraska@chemia.uj.edu.pl (L.Z.)

**Keywords:** anodic tungsten oxides, anodization, nanostructured morphology, photoelectrochemical properties

## Abstract

Although anodic tungsten oxide has attracted increasing attention in recent years, there is still a lack of detailed studies on the photoelectrochemical (PEC) properties of such kind of materials grown in different electrolytes under various sets of conditions. In addition, the morphology of photoanode is not a single factor responsible for its PEC performance. Therefore, the attempt was to correlate different anodizing conditions (especially electrolyte composition) with the surface morphology, oxide thickness, semiconducting, and photoelectrochemical properties of anodized oxide layers. As expected, the surface morphology of WO_3_ depends strongly on anodizing conditions. Annealing of as-synthesized tungsten oxide layers at 500 °C for 2 h leads to obtaining a monoclinic WO_3_ phase in all cases. From the Mott-Schottky analysis, it has been confirmed that all as prepared anodic oxide samples are n-type semiconductors. Band gap energy values estimated from incident photon−to−current efficiency (IPCE) measurements neither differ significantly for as−synthesized WO_3_ layers nor depend on anodizing conditions such as electrolyte composition, time and applied potential. Although the estimated band gaps are similar, photoelectrochemical properties are different because of many different reasons, including the layer morphology (homogeneity, porosity, pore size, active surface area), oxide layer thickness, and semiconducting properties of the material, which depend on the electrolyte composition used for anodization.

## 1. Introduction

Tungsten oxide (WO_3_) is an n-type semiconductor that has been considered so far as one of the most promising materials for photoanodes for photoelectrochemical (PEC) water splitting due to its superior charge transport properties, moderate hole diffusion length and, mostly, a relatively narrow band gap (2.5–2.8 eV). Many different methods have been employed for the synthesis of WO_3_ nanomaterials, including chemical vapor deposition (CVD) [1], hydrothermal methods [2,3], sol−gel processes [4], electrodeposition [5], anodic oxidation (anodization) [6,7,8], and many others [9]. Among these techniques, electrochemical oxidation of metallic tungsten has received considerable attention since it can be applied to synthesize nanostructured WO_3_ with various morphologies such as nanoporous [6,8,10,11,12,13,14,15] or nanotubular layers [10,16], compact films [8,12,14], nanoplates [17,18], nanowires [11], and others [11,14]. A great advantage of this method is its simplicity, versatility, and cost-effectiveness. Moreover, as-received anodic oxide films exhibit good adhesion to the conductive metallic substrate, which is another advantage in terms of its application in photoelectrochemical devices [8]. What is important, the type of the received morphology and geometrical features of the oxide film (e.g., pore/tube/wire sizes, anodic layer thickness) is strongly dependent on the conditions applied during electrolysis, in particular the electrolyte composition. For instance, nanoporous WO_3_ layers can be received during anodization of tungsten in various electrolytes containing fluoride ions [6,8,10,11,12,13,14], oxalic acid [15], and pure molten orto-phosphoric acid [19]. On the contrary, compact or almost compact oxide films can be obtained in electrolytes without fluoride ions [10,12] or when the F^−^ content is insufficient [8,12,20]. It has been also reported, that WO_3_ nanoplates can be synthesized by anodic oxidation of W in nitric acid [18] or in a mixture of sodium fluoride and sulfuric acid [17], while electrooxidation of tungsten in a NaOH solution leads to the formation of a hexagonally ordered nanobubble WO_3_ structure [21]. Moreover, all other electrosynthesis conditions such as applied voltage [8,22], electrolyte composition (especially its pH and viscosity) [10,23], temperature [8], process duration [8,10], or even hydrodynamic conditions [24], can also have a significant impact on the morphology of anodic oxide layers.

Since it is widely recognized that there is a strong correlation between the morphology and size of semiconductor and its properties, several studies comparing the photoelectrochemical and photocatalytic activity of anodic WO_3_ layers with different morphologies have been already reported [6,10,14,23,25,26]. For instance, Reyes-Gil et al. [12] have shown that the anodically formed nanoporous WO_3_ photoanodes exhibit superior photoelectrochemical performance compared to the compact ones due to the higher surface area, enhanced internal quantum yields, and effective minority−carrier diffusion lengths, consequently reducing the electron-hole recombination rate. The photoelectrochemical characterization of WO_3_ with different morphologies (nanoporous layers, nanobowls, and nanoholes) obtained by anodization of tungsten in different electrolytes has been performed by de Tacconi et al. [11], and the best photoresponse was observed for nanoporous WO_3_. On the other hand, Chin Wei Lai [10] studied the performance of WO_3_ photoanodes electrochemically synthesized in electrolytes with various F^−^ contents and confirmed an enhanced efficiency of well-developed nanotubular films under solar illumination compared to irregular nanoporous layers. Mohamed et al. [27] compared photoelectrochemical performance of WO_3_ nanoporous films with nanoflakes and found that the latter exhibit superior properties after annealing at 500 °C.

Table 1 shows a comparison of photoelectrochemical properties (photocurrent densities) of anodic tungsten oxide obtained by anodization in various electrolytes. It is clearly seen that it is difficult to compare those values because different types and intensities of light sources, supporting electrolytes, and polarization of photoanodes were used. For this reason, we propose a detailed investigation of the morphology, photoelectrochemical, and optical properties of anodic WO_3_ layers grown in different electrolytes under various operation conditions.

Despite a lot of papers discussing the influence of anodizing conditions (especially applied potential) on the morphology of anodic WO_3_ layers having already been published [8,14,19,28], detailed studies on the photoelectrochemical properties of such kind of materials grown in different electrolytes under various sets of conditions are sporadically reported in the literature. Moreover, the morphology of the photoanode is not a single factor responsible for its PEC performance. Obviously, the semiconducting/electronic properties of the material, such as a band gap, flat-band potential, dopant concentration, etc., seem to be especially important. Therefore, in this work, we report for the first time a detailed investigation of the semiconducting and photoelectrochemical properties of tungsten oxide layers obtained by anodization of metallic W in different electrolytes under various conditions. The complex characterization of the morphology and composition of as-received WO_3_ layers is also presented. A special emphasis is put on the establishment of correlations between conditions applied during anodic oxidation, morphological features of the synthesized materials, their semiconducting properties and, finally, photoelectrochemical performance of the photoanodes. 

## 2. Results

In order to compare the properties of different types of anodic WO_3_ with various morphologies, six different sets of anodizing conditions (labelled as B, C, D, G, F, Z—for details, see Table 2) were chosen on the basis of literature research and preliminary results.

**Figure 1:**
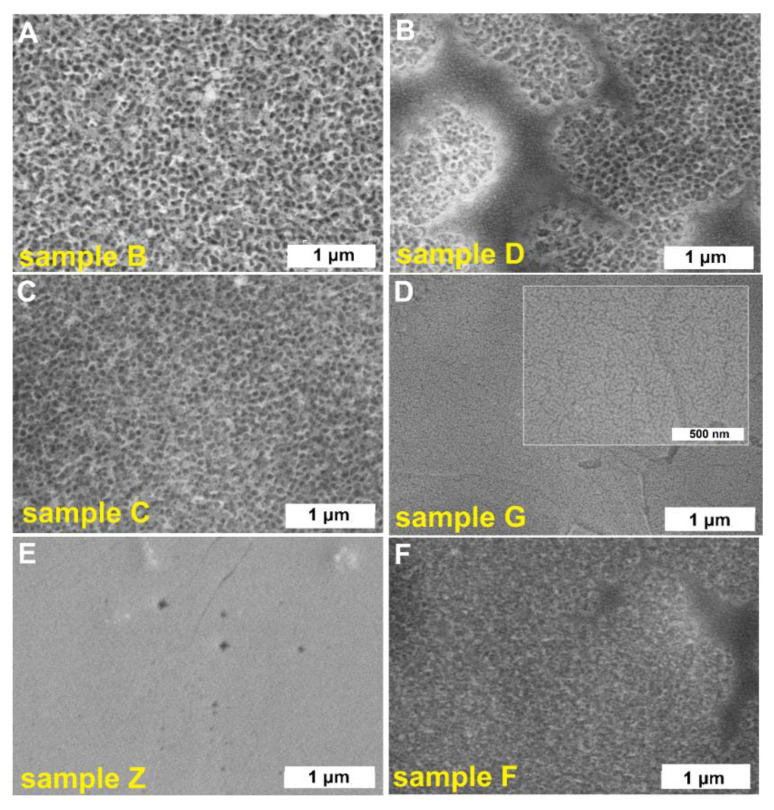
SEM images of anodic WO_3_ obtained in different anodizing conditions: 1 M(NH_4_)_2_SO_4_ + 0.075 M NH_4_F at 50 V for 4 h – sample B (**A**), 1 M Na_2_SO_4_ + 0.19 M NH_4_F at 40 V for 15 min – sample D (**B**), 1 M Na_2_SO_4_ + 0.12 M NaF at 40 V for 2 h – sample C (**C**), 0.27 M NH_4_F (in 2.2 wt.% H_2_O in ethylene glycol) at 10 V for 1 h – sample G (**D**), 1.8 M NaOH at 35 V for 45 s – sample Z (**E**), and 0.15 M NH_4_F at 30 V for 30 min – sample F (**F**).

Figure 1 shows SEM images of tungsten oxide layers obtained at different anodizing conditions. Considering aqueous electrolytes containing fluoride ions (samples B, C, D, and F), it is clear that the B-WO_3_ (Figure 1A) and C-WO_3_ (Figure 1C) layers are characterized by a well-defined nanoporous morphology. On the contrary, when the duration of the process was too short (sample F and D), anodic layers with a partially clogged porous surface were obtained (Figure 1B,F). Since it is well known that the size of the pores increases as the potential applied during anodization increases [8,14,19,28] and more uniform and smoother anodic layers are formed in viscous electrolytes [29], the anodic oxide film with smaller channels was synthesized in an ethylene glycol-based solution containing F^−^ ions and a small amount of water at the potential of 10 V (sample G, see Figure 1D). Surprisingly, contrary to the results obtained by Chen et al. [21], no oxide layers were observed on the tungsten surface after the anodization in a 1.8 M NaOH electrolyte (sample Z, Figure 1E), and this fact was confirmed by EDS results—no oxygen was found (see Figure S1, Supplementary Materials). However, very recently, Wang et al. [30] reported that efficient electrochemical polishing of tungsten can be conducted in this kind of electrolyte resulting on a smooth tungsten surface. Therefore, sample Z was not taken for further studies.

**Figure 2:**
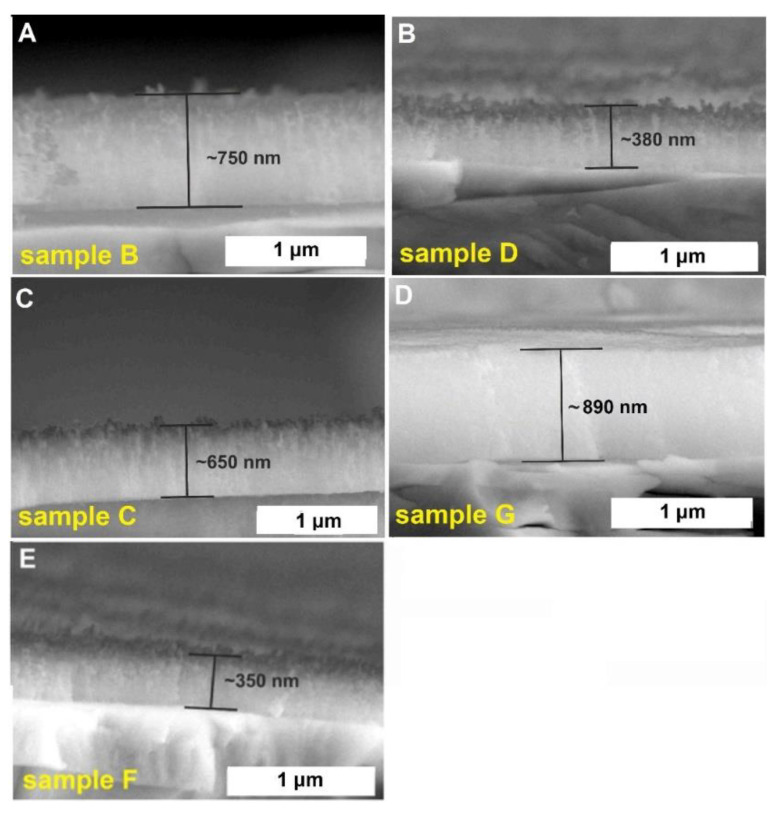
Cross-sectional SEM images of anodic WO_3_ layers obtained in different anodizing conditions: 1 M(NH_4_)_2_SO_4_ + 0.075 M NH_4_F at 50 V for 4 h - sample B (**A**), 1 M Na_2_SO_4_ + 0.19 M NH_4_F at 40 V for 15 min – sample D (**B**), 1 M Na_2_SO_4_ + 0.12 M NaF at 40 V for 2 h – sample C (**C**), 0.27 M NH_4_F (in 2.2 wt.% H_2_O in ethylene glycol) at 10 V for 1 h – sample G (**D**), 0.15 M NH_4_F at 30 V for 30 min (**E**).

Cross-sectional views of the obtained tungsten oxide films are presented in Figure 2. It is clearly visible that the received oxide layers differ in thickness, from about 400 nm (sample F) up to 890 nm (sample G). Moreover, anodic films formed in aqueous electrolytes exhibit a typical irregular rough morphology, while that grown in the ethylene glycol-based solution is uniform, more compact, and smooth (Figure 1D).

**Figure 3:**
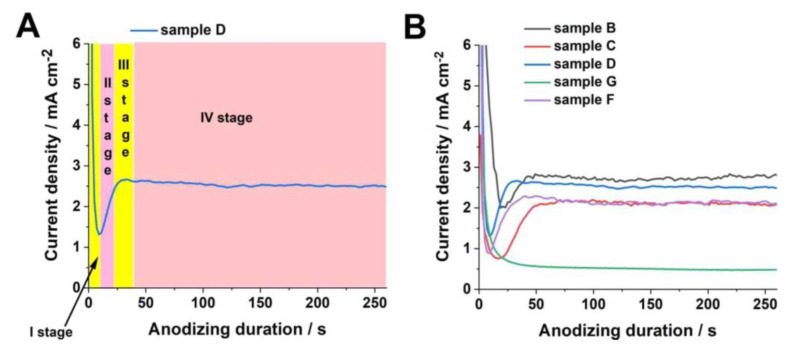
Current density vs. time curves recorded during anodic oxidation of metallic tungsten with marked different stages of anodization (**A**). Current density vs. time curves recorded during anodization of W at different conditions (**B**).

In order to study the oxide build-up process, current densities were recorded for each sample during anodization (Figure 3).

Analyzing the typical shape of current density vs. time curves recorded during anodization, it can be seen that four characteristic stages can be distinguished (Figure 3A). At the beginning of electrochemical oxidation, the surface of tungsten is covered entirely with a compact oxide layer thickening with time by a field-assisted oxide growth, which is accompanied by a significant current drop (stage I). Over the course of the process, a compact layer is transformed into initially porous as a result of the field-enhanced dissolution of anodic oxide [31] and formation of penetration paths and pore embryos in the compact oxide layer (sometimes accompanied by oxygen evolution or chemical etching of oxide with fluoride ions) [15]. Consequently, the current density increases until it reaches the maximum (stage II). At stage III, some initial pores grow up and coalesce with adjacent smaller pores, and consequently, a slight decrease in the current density with time is detected. Finally, a stable current density is observed, indicating a steady-state growth of nanostructured oxide layer (stage IV) [32]. As can be seen in Figure 3B, the typical shape of the current density vs. time cure is reproduced for all samples anodized in aqueous electrolytes. As expected, both the steady-state current density and time required to reach a local current minimum (initiation of pore formation) are strongly dependent on the anodizing conditions, especially the electrolyte composition (i.e., the higher the concentration of F^−^ ions, the earlier pore formation occurs due to more effective oxide dissolution) and applied potential (the higher the applied potential, the faster pore formation and the higher charge passing through the system) [8]. On the contrary, for anodization of the tungsten foil in the ethylene glycol-based solution (sample G), the current density decreases continuously up to ca. 50 s when a stable value is reached. Such a shape of the current-time curve without a local minimum is typical for the formation of compact anodic layers, which is strongly in line with the morphology of sample G shown in Figure 1D and Figure 2D.

**Figure 4:**
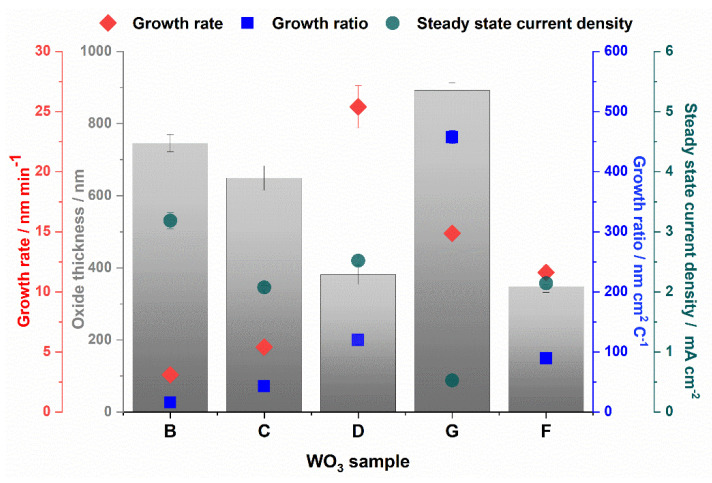
The oxide growth rate (red), growth ratio (blue), steady state current density (green), and oxide thickness of WO_3_ layers obtained in different anodizing conditions.

The steady-state current density and growth rate as well as a growth ratio, defined as the average oxide thickness divided by the charge density, were calculated for all studied WO_3_ samples, and the results are collected in Figure 4. 

Among the samples anodized in aqueous solutions, the highest growth ratio and growth rate were observed for the shortest duration of anodization process (sample G). In our recent work [8], we confirmed that the most effective oxide thickening is observed at the initial stage of anodization, and the longer the process, the more effective is the chemical etching of the oxide film caused by F^−^ ions. For detailed analysis of the influence of anodizing parameters on the growth rate and efficiency of WO_3_ formation during anodization in aqueous electrolytes containing fluorides, please refer to our previous paper [8]. For the WO_3_ layer received in the ethylene glycol based electrolyte at 10 V (sample G), the growth ratio reaches a much higher value (460 nm cm C^−1^) compared with other samples anodized at higher potentials in aqueous electrolytes (15–120 nm cm C^−1^). The highest efficiency of the oxide formation at the proposed conditions is a result of both a much slower oxide etching by F^−^ ions in the non-aqueous electrolyte [33] and less effective field-assisted oxide dissolution caused by a weaker electric field. 

**Figure 5:**
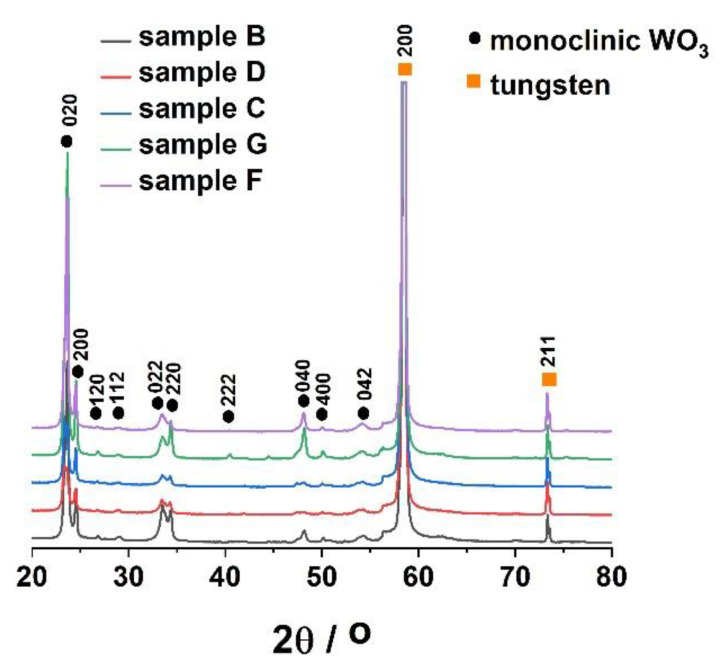
X-ray diffraction (XRD) patterns of WO_3_ layers obtained in various anodizing conditions and annealed in air at 500 °C for 2 h.

The as-received anodic WO_3_ samples were then subjected to controlled annealing treatment in air at 500 °C for 2 h [34]. Afterward, all materials were characterized by X-ray diffraction. As can be seen in Figure 5, planes that can be assigned to the metallic substrate ((200) and (211)) and monoclinic tungsten trioxide ((020), (200), (120), (112), (022), (220), (222), (040), (400), and (042)) can be clearly distinguished in the XRD patterns of anodic oxides. 

**Figure 6:**
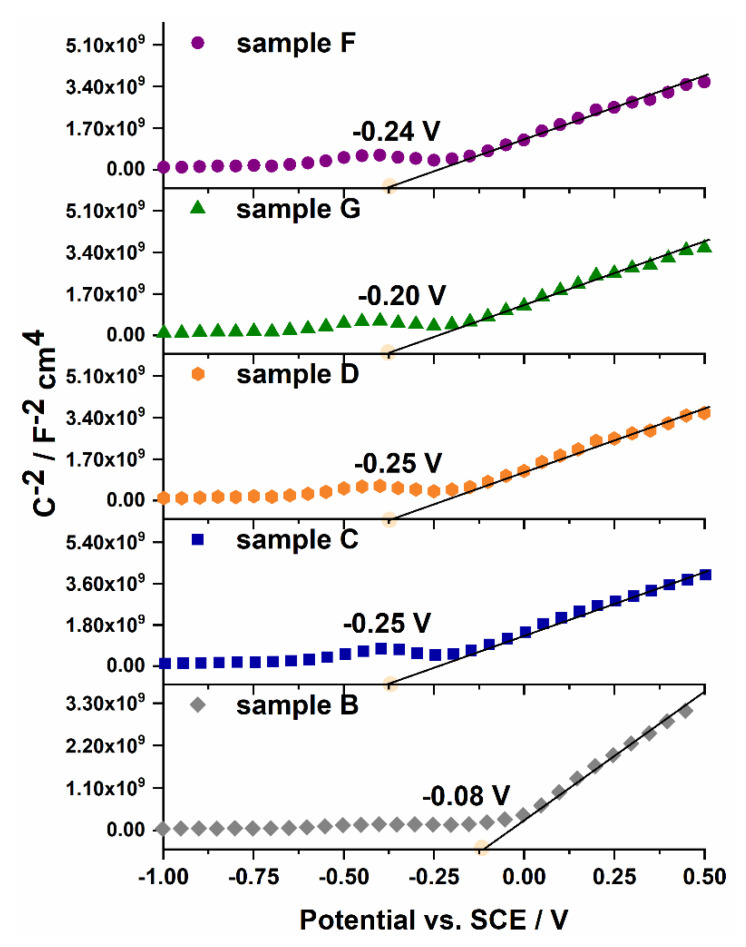
Mott-Schottky analyses performed at 1000 Hz in 0.1 M KNO_3_ electrolyte measured for all tested anodic WO_3_ samples.

Semiconducting properties of the obtained anodic tungsten oxide layers were studied using Mott-Schottky analysis (1) [35,36,37]:

(1) $$C_{SC}^{-2}=\left(\frac{2}{{\varepsilon \varepsilon }_{0}qN_{d}}\right)\left(E-E_{fb}-\frac{kT}{q}\right)$$

where *C_sc_* is the capacitance of the space charge region (F cm^−2^), *N_d_* is donor density (cm^−3^), *ε* is the dielectric constant of porous tungsten oxide (20) [36,38], *ε_0_* is permittivity of free space (8.85 × 10^−14^ F cm^−1^), *q* is the electron charge (1.602 × 10^−19^ C), *E* is the applied potential (V), *E_fb_* is a flat band potential (V), *T* is the absolute temperature (K), and *k* is the Boltzmann constant (1.38 × 10^−23^ J K^−1^). The Mott−Schottky analysis allows probing the semiconductor/electrolyte interface by capacitance-voltage measurements, and estimates the donor density and flat band potential of semiconducting material. The dependence *C_sc_* on the potential was recorded for all studied samples at the frequency of 200, 500, and 1000 Hz. As can be seen in Figure 6, a positive slope of linear part of the curves indicates an n-type semiconducting behavior of all prepared tungsten oxide layers. The estimated flat band potentials are negative for all studied samples and vary from −0.08 V to −0.25 V vs. SCE (see Table 3). As can be seen, the flat band potential for sample B is slightly more positive than for other samples and might indicate an improved photoelectrochemical properties over the other anodic oxides [39].

In a similar way, flat band potentials were determined from the intercepts of the linear parts of the Mott-Schottky curves measured at different frequencies (for details, see Table S1, Supplementary Materials). Slight differences in the estimated values may result from the porosity and non-homogeneity of the tested surfaces. This effect is often observed for crystalline porous materials [35,40]. The donor densities were also calculated for all tested materials, and the obtained values are collected in Table 3. In general, tungsten oxide-based materials described in the literature exhibit donor densities in the range of 10^19^–10^22^ cm^-3^, depending on the synthesis method. The values typically reported for oxide layers obtained by anodization (10^22^ cm^−3^) [24,41,42,43,44,45] are in agreement with those obtained for the anodic WO_3_ samples studied in this work. The highest values were received for samples B and G. 

**Figure 7:**
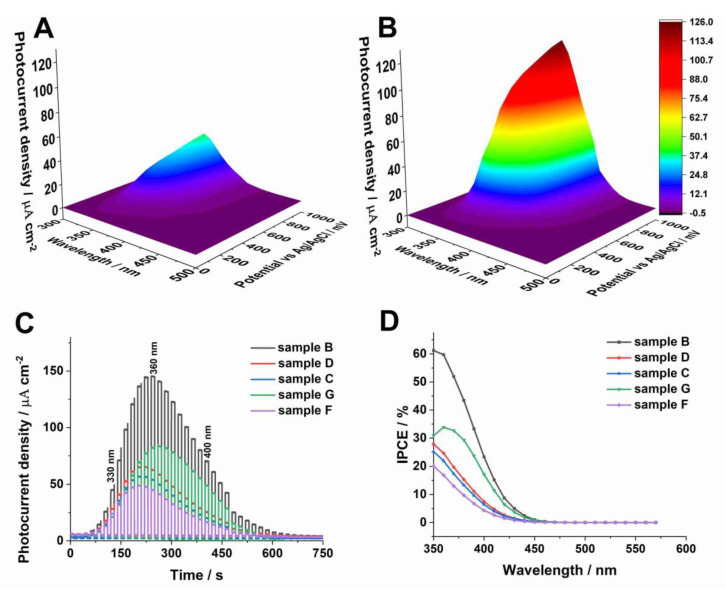
Photocurrent density as a function of the incident light wavelength and applied potential recorded in 0.1 M KNO_3_ for the anodized and annealed sample F (**A**) and B (**B**). The photoelectrochemical response of all studied WO_3_ samples at 1 V vs. Ag/AgCl (**C**) and corresponding IPCE spectra (**D**).

Photoelectrochemical properties of the anodic WO_3_ samples obtained at different conditions were also studied and the results are presented in Figure 7. The photocurrent maps, showing photocurrent densities as a function of incident light wavelength and applied potential, were recorded for all studied photoanodes. Typical 3D spectra with the lowest (sample F) and highest (sample B) photoresponse of anodic WO_3_ are shown in Figure 7A,B, respectively. As can be seen, the highest photocurrents were generated when the photoanodes were polarized with the potential of 1 V vs. SCE. Therefore, the photoelectrochemical response of all tested materials during a sequential illumination with the wavelength in the range of 300–500 nm was examined under the same conditions (Figure 7C). It is clear that the maximum photocurrent density was observed for sample B (140 µA cm^−2^ at 350 nm). It can be attributed to a higher donor density and consequently a higher conductivity of the material (lower donor densities result in a significant decline in resulting photocurrents).

Based on the photoelectrochemical measurements, the incident photon to current efficiency (IPCE) values were calculated using the following equation (2) [34,46]:

(2) $$\mathrm{IPCE}=1240\cdot \frac{I_{p}\left(\lambda \right)}{P\left(\lambda \right)\lambda }$$

where *I_p_*(λ)—photocurrent density [A m^−2^] at the wavelength λ (nm), *P*(λ)—incident power density of light [W m^−2^] at the wavelength λ (nm), 1240—constant [W nm A^−1^]. The obtained IPCE spectra are collected in Figure 7D. As can be seen, the highest IPCE value (c.a. 61% at the wavelength of 350 nm) was observed for sample B whereas other anodic materials exhibit twice-lower values.

**Figure 8:**
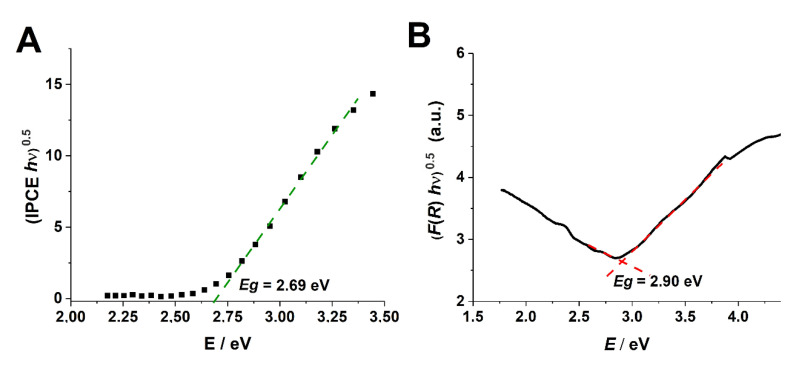
Band gap energy of sample B determined from (IPCE *h*ν)^0.5^ vs. *h*ν (**A**) and (*F*(*R*) *h*v)^0.5^ vs. *h*v (**B**) curves.

In order to better characterize semiconducting properties of anodic tungsten oxides, average band gap energies (*E_g_*) were determined from (IPCE *h*ν)^0.5^ vs. *h*ν curves (an example is shown in Figure 8A), since it is known that WO_3_ possesses an indirect band gap [7,47]. The optical band gaps of anodic WO_3_ layers were also estimated from UV-Vis diffusion reflectance measurements (for details, see Figure S2, Supplementary Materials) using the Tauc method (see Figure 8B). The average band gap energies determined by both methods are collected in Table 2. As can be seen, the band gap values are in the range between 2.7–3.0 eV, and no significant influence of anodizing conditions such as the electrolyte composition, time, and applied potential on the band gap was found. 

## 3. Discussion

**Figure 9:**
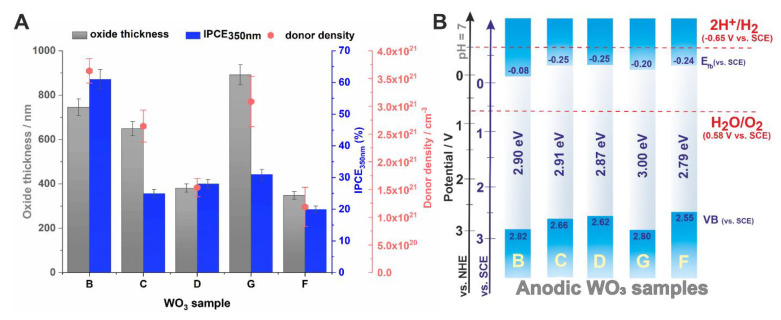
The correlation between oxide layer thickness, obtained IPCE value at 350 nm, and potential of 1 V vs. SCE and donor densities for anodic WO_3_ materials (**A**). Energy diagrams for all tested anodic WO_3_ samples **(B**).

Based on research conducted for the tested WO_3_ samples, it can be stated that operating conditions applied during the anodization process are of great importance in designing photoanodes with enhanced properties. Although the estimated band gaps do not differ significantly within the samples, their photoelectrochemical properties can be very different. It is related with a combination of several important factors such as the morphology of anodic oxide (homogeneity, porosity, pore size, active surface area), oxide layer thickness, and mostly, properties of the semiconductor itself (e.g., density of charge carriers), which in turn depend on anodizing conditions, including electrolyte composition. As mentioned before, the porosity of anodic oxide has a significant effect on its photoelectrochemical properties. Consequently, porous structures with a larger active surface area exhibit better photoelectrochemical performance due to a reduced rate of electron−hole recombination. Moreover, nanoporous oxide layers can release more photoinduced electron−hole pairs compared to compact materials [6,10,12,48,49]. It can explain a significantly worse photoresponse of sample G (more compact) compared to sample B (much more porous), while for both samples, the layer thickness and donor density are similar (see Figure 9A).

Another aspect worth mentioning is thickness of the semiconducting layer. When the thickness of anodic oxide increases to optimal value, the photocurrent density increases because of the greater number of photogenerated electron-hole pairs. However, for the thicker oxide layers (thicker than the optimal thickness), the photoresponse worsens due to a limited depth of pore penetration by the incident light [50] and possible recombination of photogenerated charge carriers during their migration through the oxide layer (towards the current collector) over longer distances [50,51,52]. Therefore, the formation of thick anodic WO_3_ films (samples C and D) is not an effective strategy for improvement of the photoanode performance. For instance, sample C being almost twice as thick as sample D and having a better developed porous morphology (see Figure 1) generates even lower photocurrents (Figure 9A). Comparing the IPCE values obtained for samples having similar thicknesses and well-defined porous morphology (i.e., samples D and F), the better performance of sample D can be explained in terms of the higher *N_d_* value (Figure 9A). In order to sum up the semiconducting properties of the investigated materials, the energy diagrams were constructed (Figure 9B) based on the assumption that for n-type semiconductors, the flat band potential merges practically with the conduction band edge [34,53].

The superior photoelectrochemical properties of sample B are a direct consequence of its optimal morphology (mainly a well-developed porous surface) combined with electronic properties (high donor density). Finally, in order to assess the stability of photoanode response over time, sample B (showed the best photoelectrochemical performance) was tested for 10 weeks (for details, see Figure S3, Supplementary Materials). The average IPCE value calculated for consecutive 10 measurements performed at 350 nm and polarization 1 V vs. SCE was about 58.5 ± 2.8%. The obtained results suggest that the photoanode exhibits very stable performance in terms of the generated photocurrent. 

## 4. Materials and Methods

### 4.1. Preparation of Anodic WO_3_ Layers

Tungsten oxide layers were obtained by single-step anodic oxidation of metallic tungsten (99.95%, 0.2 mm thick, Goodfellow, Huntingdon,, England) carried out in different electrolytes. Applied electrooxidation conditions, including the electrolyte composition, applied voltage, and duration of the process are collected in Table 4. Anodizations were carried out in a two-electrode cell, where the W foil was used as an anode and the Pt mesh as a cathode. All syntheses were performed at a constant temperature of 20 °C in a continuously stirred (250 rpm) electrolyte [8]. In order to obtain a photoactive phase, the as-received samples were subjected to annealing in air at 500 °C for 2 h (heating rate of 2 °C min^−1^) using a muffle furnace (FCF 5SHM Z, Czylok, Krakow, Poland) [34].

### 4.2. Characterization of Anodic WO_3_ Layers

The morphology of the obtained materials was verified using a field emission scanning electron microscope (FE-SEM/EDS, Hitachi S-4700 with a Noran System 7, Tokyo, Japan. The thickness of the anodic films was estimated directly from SEM images by using WSxM image processing software [54]. The phase composition of received samples was determined using the X-ray diffractometer Rigaku Mini Flex II (Rigaku, Tokyo, Japan) with monochromatic Cu Kα radiation (λ = 1.5418 Å) at the 2θ range of 20–80°. The diffuse reflectance spectra of the samples were recorded in the range of 250–800 nm at room temperature using the Perkin Elmer Lambda 750S UV/Vis/NIR spectrophotometer (Waltham, MA, USA).

### 4.3. Electrochemical and Photoelectrochemical Measurements

All photoelectrochemical tests were carried out in a Teflon cell with a quartz window in a three-electrode system, where anodic tungsten oxide layers were used as working electrodes (WE), a platinum foil as a counter electrode (CE), and Ag/AgCl/KCl (3 M KCl) electrode as a reference electrode (RE). The generated photocurrents were measured in 0.1 M KNO_3_ using a photoelectric spectrometer equipped with the 150 W xenon arc lamp (Instytut Fotonowy, Krakow, Poland) and combined with a potentiostat (Instytut Fotonowy, Poland). The Mott-Schottky analysis was carried out using the Gamry Reference 3000 potentiostat (Warminster, PA, USA ) at frequencies of 200, 500, and 1000 Hz and DC potential range 0–1 V.

## 5. Conclusions

In summary, a detailed investigation of the anodic formation of tungsten oxide layers in different electrolytes confirmed that the morphology of anodic oxide depends strongly on anodizing conditions, especially the electrolyte composition. The n-type semiconducting behavior of all obtained tungsten oxides was confirmed by Mott-Schottky analyses. Despite the fact that no significant effect of anodizing parameters on the band gap value was observed, the other semiconducting properties, including flat band potential and, especially, donor densities were found to be strongly dependent on the conditions applied during anodic oxidation. In consequence, the studied samples exhibited different photoelectrochemical properties because of several important reasons, including differences in the surface morphology (homogeneity, porosity, pore size, active surface area), oxide layer thickness, and aforementioned semiconducting properties. Therefore, it should be emphasized that not only the morphology of the resulting sample should be taken into consideration when looking for optimal conditions for the fabrication of the most promising anodic WO_3_ photoanode, since the electrolysis parameters also affect the semiconducting nature of the nanostructured film itself. Here, we found that WO_3_ with a well-defined porous morphology and the best PEC properties can be formed by anodization in 1 M (NH_4_)_2_SO_4_ and 0.075 M NH_4_F at 50 V during 4 h followed by annealing in air at 500 °C. Importantly, the obtained photoanode exhibited very stable photoelectrochemical performance over 10 weeks. 

We expect that the as-prepared tungsten oxide sample can be a promising material for further investigations, such as doping or creating heterojunctions to shift photoresponse into visible light range. Moreover, the presented differences in semiconducting properties of anodic materials might be beneficial for other applications of anodic tungsten oxide layers, including sensors, photocatalysts, and smart windows.

## Figures and Tables

**Table 1:** Structural features and photoelectrochemical properties of anodic WO_3_ formed in different electrolytes.

Electrolyte Composition; Time of Anodization; Applied Voltage	Morphology; Oxide Thickness	Current Density (at a Given Potential)	Electrolyte	Light Source and Intensity	Ref.
0.15 M NH_4_F (glycerol/water 50/50 vol %); 1 h; 40 V	Nanotubes	0.38 mA cm^−2^ (0.6 V vs. SCE)	0.5 M Na_2_SO_4_, 25 vol % methanol	LED (15 mW cm^−2^)	[7]
1 M HNO_3_; 1 h; 40 V	Nanoflakes	1.17 mA cm^−2^ (1.2 V vs. SCE)	1 M H_2_SO_4_	Xe lamp (AM 1.5 G filter; 100 mW cm^−2)^	[27]
10 wt% K_2_HPO_4_/glycerol; 20 h; 50 V	Mesoporous layers; 2.5 μm	~1.4 mA cm^−2^ (1.0 V vs. Ag/AgCl)	1 M HClO_4_	Xe lamp (AM1.5 filter)	[26]
0.1 M NaF; 24 h; 60 V	Porous film; 2. 59 μm	0.75 mA cm^−2^ (1.23 V vs. RHE)	0.1 M HCl	Xe lamp (100 mW cm^−2^)	[12]
0.15 M NaF; 1 h; 60 V	Nanoporous	3.21 mA cm^−2^ (2.0 V vs. Ag/AgCl)	0.5 M Na_2_SO_4_	Xe lamp	[14]
0.15 M NaF; 1 h; 60 V	Nanoporous	0.63 mA cm^−2^ (2.0 V vs. Ag/AgCl)	0.5 M Na_2_SO_4_	Xe lamp	[11]

**Table 2:** Band gap values (eV) of anodic WO_3_ layers obtained in various anodizing conditions and then annealed in air at 500 °C for 2 h estimated from IPCE and UV-Vis reflectance measurements.

Anodization Conditions	WO_3_ Sample Label	Photoelectrochemical Measurements	UV-Vis Diffuse Reflectance Spectroscopy Measurements
1 M (NH_4_)_2_SO_4_ and 0.075 M NH_4_F; 50 V; 240 min	B	2.69 ± 0.05	2.90 ± 0.06
1 M Na_2_SO_4_ and 0.12 M NaF; 40 V; 120 min	C	2.71 ± 0.05	2.91 ± 0.06
1 M Na_2_SO_4_ and 0.19 M NH_4_F; 40 V; 15 min	D	2.72 ± 0.05	2.87 ± 0.06
0.27 M NH_4_F in 2.2 wt.% H_2_O in ethylene glycol; 10 V; 60 min	G	2.68 ± 0.05	3.00 ± 0.06
0.15 M NH_4_F; 30 V; 30 min	F	2.74 ± 0.05	2.79 ± 0.06

**Table 3:** Estimated flat band potentials (*E_fb_*), donor densities (*N_d_*) of WO_3_ obtained in various anodizing conditions and annealed in air at 500 °C for 2 h.

Anodization Conditions	WO_3_ Sample Label	*E_fb_* vs. SCE / V	*N_d_* / cm^−3^
1 M (NH_4_)_2_SO_4_ and 0.075 M NH_4_F; 50 V; 240 min	B	−0.08	(3.64 ± 0.22) × 10^21^
1 M Na_2_SO_4_ and 0.12 M NaF; 40 V; 120 min	C	−0.25	(2.64 ± 0.29) × 10^21^
1 M Na_2_SO_4_ and 0.19 M NH_4_F; 40 V; 15 min	D	−0.25	(1.53 ± 0.17) × 10^21^
0.27 M NH_4_F in 2.2 wt.% H_2_O in ethylene glycol; 10 V; 60 min	G	−0.20	(3.08 ± 0.45) × 10^21^
0.15 M NH_4_F; 30 V; 30 min	F	−0.24	(1.18 ± 0.35) × 10^21^

**Table 4:** Operating Conditions of Anodic Oxidation of Tungsten Foil.

Electrolyte Composition	Time of Anodization / min	Applied Voltage / V	WO_3_ Sample Label
1 M (NH_4_)_2_SO_4_ + 0.075 M NH_4_F	240	50	B
1 M Na_2_SO_4_ + 0.12 M NaF	120	40	C
1 M Na_2_SO_4_ + 0.19 M NH_4_F	15	40	D
0.27 M NH_4_F + 2.2 wt.% H_2_O, ethylene glycol based solution	60	10	G
0.15 M NH_4_F	30	30	F
1.8 M NaOH	45 s	35	Z

## Supplementary Materials

Supplementary Materials are available online, Figure S1: EDS spectra of tungsten foil and tungsten sample anodized in a 1.8 M NaOH solution, Figure S2. UV-Vis reflectance spectra for all studied WO_3_ samples after anodization and annealing in air at 500 oC for 2 h, Figure S3. IPCE values obtained at 1 V vs. SCE for the sample B over 10 weeks of storage with corresponding average response, Table S1. Flat band potentials estimated for all studied WO_3_ samples at 200, 500 and 1000 Hz.
