# Supplementary material for: Improving Photoelectrochemical Properties of Anodic WO3 Layers by Optimizing Electrosynthesis Conditions
Source: Molecules. 2020 Jun 25;25(12):2916. doi: 10.3390/molecules25122916 (PMC7357059; doi:10.3390/molecules25122916)
Supplement: Supplementary file 1 [file molecules-25-02916-s001.pdf]

Improving photoelectrochemical properties of anodic WO_3 layers by optimizing electrosynthesis conditions

Marta Zych, Karolina Syrek*, Leszek Zaraska, Grzegorz D. Sulka*

Jagiellonian University, Faculty of Chemistry, Department of Physical Chemistry and Electrochemistry, Gronostajowa 2, 30-387 Krakow, Poland

***** Corresponding author. E-mail: syrek@chemia.uj.edu.pl (K.S.), sulka@chemia.uj.edu.pl (G.D.S)

Jagiellonian University, Faculty of Chemistry, Department of Physical Chemistry & Electrochemistry

Gronostajowa 2, 30387 Krakow, Poland

Tel: +48 12 686 27 70

Fax: +48 12 686 27 50

Keywords: anodic tungsten oxides; anodization; nanostructured morphology; photoelectrochemical properties

1. EDS spectra

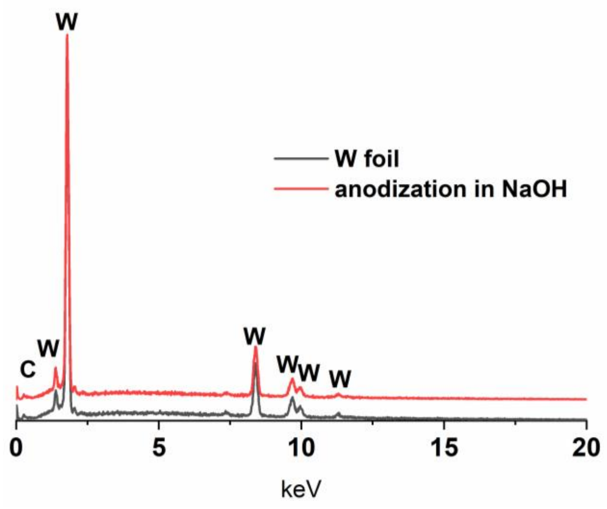

Fig. S1. EDS spectra of tungsten foil and tungsten sample anodized in a 1.8 M NaOH solution.

2. Mott-Schottky analysis

Table. S1. Flat band potentials estimated for all studied WO_3 samples at 200, 500 and 1000 Hz.

WO_3 sample label	Flat band potential / V vs. SCE		
	200 Hz	500 Hz	1000 Hz
**B**	-0.05	-0.03	-0.08
**C**	-0.10	-0.15	-0.25
**D**	-0.15	-0.20	-0.25
**G**	-0.12	-0.17	-0.20
**F**	-0.10	-0.18	-0.24

3. UV-Vis reflectance measurements

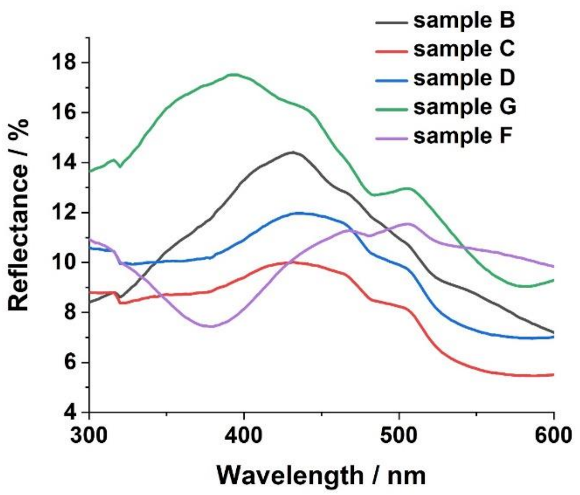

Fig. S2. UV-Vis reflectance spectra for all studied WO_3 samples after anodization and annealing in air at 500 ^{o}C for 2 h.

The optical band gaps of anodic WO_3 layers were also estimated from UV-Vis diffusion reflectance measurements using the Tauc method (see Fig. 8B). The method is based on the assumption that the absorption coefficient α depends on energy according to the following relation (1):

$$(\alpha \cdot h\nu)^{1/\gamma} = B \cdot (h\nu - E_g) \qquad (1)$$

where h – the Planck constant, ν – frequency of photon, B – constant depending on the material disorder, E_g – the band gap energy, γ – constant depending on the nature of electron transition, which is equal to ½ for direct and 2 for indirect transition band gaps [3, 54]]. The recorded reflectance spectra were transformed to absorption spectra using the Kubelka-Munk function given by the equation (2):

$$F(R_\infty) = \frac{(1-R_\infty)^2}{2R_\infty} \qquad (2)$$

where: R_∞– reflectance, F(R_∞) – Kubelka-Munk function [54 – 56]. Since F(R_∞) is proportional to α, in the next step α in equation 3 can be substituted with F(R_∞) [54 – 56].

4. Photoelectrochemical response stability

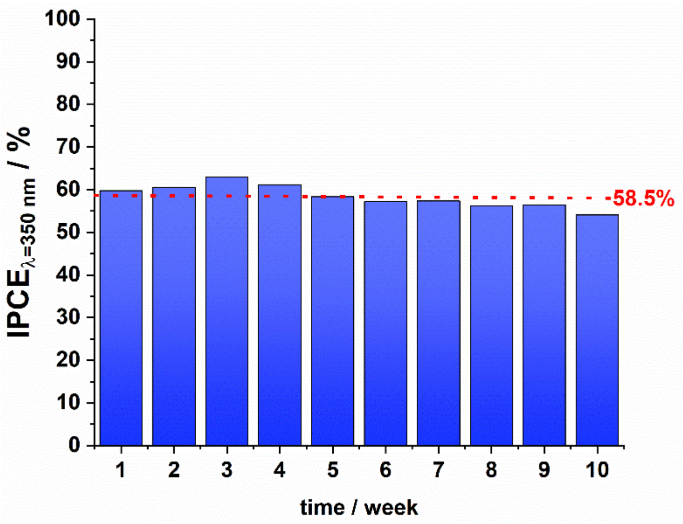

Fig. S3. IPCE values obtained at 1 V vs. SCE for the sample B over 10 weeks of storage with corresponding average response.
